# Epidermal growth factor receptor mutation in combination with expression of MIG6 alters gefitinib sensitivity

**DOI:** 10.1186/1752-0509-5-29

**Published:** 2011-02-18

**Authors:** Yoshimi Naruo, Takeshi Nagashima, Ryoko Ushikoshi-Nakayama, Yuko Saeki, Takashi Nakakuki, Takashi Naka, Hiroshi Tanaka, Shih-Feng Tsai, Mariko Okada-Hatakeyama

**Affiliations:** 1 Laboratory for Cellular Systems Modeling, RIKEN Research Center for Allergy and Immunology (RCAI), 1-7-22 Suehiro-cho, Tsurumi-ku, Yokohama, Kanagawa 230-0045, Japan; 2 Department of Systems Biology, Bioinformatics, Graduate School of Biomedical Science, Tokyo Medical and Dental University, 1-5-45 Yushima, Bunkyo-Ku, Tokyo 113-8510, Japan; 3 Department of Mechanical Systems Engineering, Faculty of Engineering, Kogakuin University, 1-24-2 Nishi-Shinjuku, Shinjuku-ku, Tokyo 163-8677, Japan; 4 Department of Information Science, Faculty of Information Science, Kyushu Sangyo University, 2-3-1 Matsukadai, Higashi-ku, Fukuoka 813-8503, Japan; 5 Division of Molecular and Genomic Medicine, National Health Research Institutes, 35 Keyan Road, Zhunan, Miaoli 350, Taiwan

## Abstract

**Background:**

Epidermal growth factor receptor (EGFR) signaling plays an important role in the regulation of cell proliferation, survival, metastasis, and invasion in various tumors. Earlier studies showed that the EGFR is frequently overexpressed in non-small-cell lung cancer (NSCLC) and EGFR mutations at specific amino acid residues in the kinase domain induce altered responsiveness to gefitinib, a small molecule EGFR tyrosine kinase inhibitor. However, the mechanism underlying the drug response modulated by EGFR mutation is still largely unknown. To elucidate drug response in EGFR signal transduction pathway in which complex dynamics of multiple molecules involved, a systematic approach is necessary. In this paper, we performed experimental and computational analyses to clarify the underlying mechanism of EGFR signaling and cell-specific gefitinib responsiveness in three H1299-derived NSCLC cell lines; H1299 wild type (H1299WT), H1299 with an overexpressed wild type EGFR (H1299EGFR-WT), and H1299 with an overexpressed mutant EGFR L858R (H1299L858R; gefitinib sensitive mutant).

**Results:**

We predicted and experimentally verified that Mig6, which is a known negative regulator of EGFR and specifically expressed in H1299L858R cells, synergized with gefitinib to suppress cellular growth. Computational analyses indicated that this inhibitory effect is amplified at the phosphorylation/dephosphorylation steps of MEK and ERK.

**Conclusions:**

Thus, we showed that L858R receptor mutation in combination with expression of its negative regulator, Mig6, alters signaling outcomes and results in variable drug sensitivity.

## Background

The ErbB family receptors belong to the receptor tyrosine kinases (RTKs) and consist of four members; ErbB1 (also known as EGFR; epidermal growth factor receptor), ErbB2, ErbB3 and ErbB4 [1-4]. EGFR is distributed various tissues of the human body [5-7], and plays a critical role in the regulation of a variety of cellular responses ranging from cell differentiation, growth, proliferation, apoptosis, migration and adhesion [2,8].

EGFR is frequently overexpressed in various human tumors including non-small-cell lung cancer (NSCLC) and is associated with poor outcome [9,10]. In many cases, enhanced EGFR signaling leads to abnormal cellular processes and often induces cancer [11,12]. Certain NSCLC patients have mutations at specific amino acid residues in the kinase domain of EGFR and show altered responsiveness to gefitinib (Iressa), an EGFR tyrosine kinase inhibitor. The L858R substitution (an arginine for leucine substitution at amino acid 858) is one of the most frequently reported mutations [13] and shows good responses to gefitinib [14-16]. It was reported that the L858R mutation enhances gefitinib sensitivity due to a structural change in the kinase domain resulting in an increased binding affinity of gefitinib for its ATP binding pocket *in vitro *[16]. On the other hand, a large scale binding assay using different types of kinases showed that the difference in binding affinity of the EGFR itself may not have a great effect on gefitinib sensitivity [17]. Based on these observations, we speculated that other unknown factors affect gefitinib sensitivity *in vivo *rather than alteration of the binding affinity. So far, cells with the L858R-mutated EGFR have been reported to have two characteristics. First, Mig6 (mitogen-inducible gene 6) is highly expressed in the L858R-mutated EGFR cells [18]. Mig6 is an adaptor molecule that binds to an activating kinase domain of an EGFR [19] and functions as a negative regulator of EGFR kinase [19-21]. Mutation and downregulation of Mig6 are often observed in human lung cancer cell lines [22] and also correlate with a reduced survival rate in breast cancer patients [23,24]. Secondly, ubiquitin-dependent EGFR degradation mediated by Cbl is enhanced in the L858R cells [15]. Both of these two characteristics seem to contribute to the negative regulation of the EGFR signaling pathway. However, no mechanistic explanation has been found for the contributions of these molecules to the gefitinib sensitivity of the L858R mutation.

Recent studies showed that dynamics and regulation of the intracellular signaling cascades are efficiently elucidated with an assistance of computational simulations [25-37]. To obtain a logical understanding of the gefitinib sensitivity associated with L858R mutation, the mathematical analysis of the EGFR signaling pathway should be more preferable rather than sole experimental representations.

In this study, we used experimental and computational approaches to investigate regulatory mechanisms that distinguish cell-specific gefitinib sensitivity in H1299 human NSCLC cell lines. We have modified the existing kinetic model of the EGFR signaling pathway and built new models for H1299 wild type (H1299WT), H1299 with overexpressed wild type EGFR (H1299EGFR-WT), and H1299 overexpressing the EGFR with L858R mutation (H1299L858R). The three types of cells showed different signaling dynamics in response to EGF stimulation. Overexpression of wild type EGFR induced high and sustained phosphorylation of EGFR, Shc, MEK (mitogen-activated protein kinase kinase) and ERK (extracellular signal-regulated kinase), while the L858R mutation reduced these response levels. In addition, H1299L858R, which is supposed to be more sensitive to gefitinib than H1299EGFR-WT, was effectively inhibited by gefitinib administration at the downstream part of the signaling pathway (MEK and ERK) compared with H1299EGFR-WT, but, surprisingly, not at the upstream part of the pathway (EGFR and Shc). The model incorporated Mig6, but not Cbl overexpression, successfully captured the signaling behavior observed in our experimental data, implying that Mig6 is responsible for enhancing gefitinib sensitivity. Detailed computational analyses revealed that Mig6 amplifies gefitinib sensitivity at the steps of MEK phosphorylation/dephosphorylation and ERK phosphorylation/dephosphorylation. We experimentally verified that overexpression of Mig6 contributed to suppressing cellular growth in the presence of gefitinib. Our analysis further suggested that the combination of Mig6 and gefitinib exhibits a synergistic effect in inhibiting EGFR signaling pathway.

## Methods

### Cell culture

H1299 human lung cancer derivatives, H1299WT, H1299EGFR-WT and H1299L858R, were established as described elsewhere [15]. Cells were maintained in RPMI1640 medium supplemented with 10% fetal bovine serum and 1 mM sodium pyruvate. Prior to growth hormone treatment, the cells were serum-starved for 16-24 hours. For the EGFR kinase inhibition, gefitinib (a generous gift from Astra Zeneca, UK) was added 20 minutes prior to the growth factor treatment. The cells were incubated with 10 nM of EGF for 1, 5, 10, 30, 120 and 360 minutes and then washed two times with phosphate buffered saline (PBS) and lysed with Bio-Plex lysis buffer (Bio-Rad laboratories, Hercules, CA). Cell lysates were cleared by centrifugation, and the total protein concentration of the supernatant was determined using a protein assay reagent (Bio-Rad laboratories) and analyzed by western blot. Cells that were not treated with growth hormone were used as the control.

### Western blot analysis

SDS-PAGE and membrane transfer were performed using standard protocols. Antibodies against anti-phospho-EGFR (PY1068), doubly phosphorylated p44/42 ERK (Thr202/Tyr204), ERK, phospho-MEK1/2 (Ser217/221), MEK, Mig6 and actin were purchased from Cell Signaling Technology, Inc. (Beverly, MA). Anti-phospho-Shc (Tyr317), anti-Shc antibodies and anti-EGFR antibodies were purchased from Upstate Biotechnology (Lake Placid, NY). Protein band intensities were quantified using a densitometer (Fuji Film Corp., Japan). Normalization procedure is described in earlier study [33]. Briefly, the maximum value of protein phosphorylation level among three cell lines (for example, phospho-EGFR at 10 min in EGFR-WT cells) was set to 1 and the values at t = 0 minutes were set to 0 under the assumption that all the proteins were inactive before EGF stimulation. We considered that total protein level of EGFR is equal in EGFR-WT and L858R cells. All concentrations of Shc, MEK and ERK were considered to be equal in three cell lines (Additional file 1, Figure S1).

### Mig6 overexpression

The *MIG6 *gene was amplified from a human ERRFI1 cDNA purchased from OriGene (Rockville, MD) using the primers; *MIG6 *Forward 5' GCT TGT CGA CTC TAG AGA TGT CCC AGA ATA AGG CAC AAT G-3' and *MIG6 *Reverse 5'-GCG GCC GCA ATC TAG ATC TGC TGA ACC ATG ACC CCA AG-3'. The resulting DNA fragment was cloned into the vector pCMV-6-Neo (OriGene) using the *Xba I *restriction site. Cells were seeded in 96 well plates at 1 × 10^5 ^cells/well. Transfection of the *MIG6 *gene was performed using the Lipofectamine LTX (Invitrogen, Carlsbad, CA) and CombiMAG magnetofection kit (Chemicell GmbH, Berlin, Germany) according to manufacturer's protocol. Control cells were transfected with pCMV-6-Neo vector. After 8 hours of transfection, cells were supplemented with serum free RPMI1640 media. The following day, cells were treated with 10 nM EGF in the presence or absence of gefitinib.

### Cell Viability Assay

Cell viabilities of H1299 cells were measured by an MTT (3-[4,5-dimethylthiazol-2-yl]-2,5-diphenyltetrazolium bromide) cell proliferation assay 3 days after stimulation with or without 10 nM EGF in the presence of various doses of gefitinib (0, 0.1, 0.5, 1, and 5 μM) using the Cell Count Kit SF (Nacalai Tesque, Kyoto, Japan). The cell viability was determined by optical density (OD) at 450 nm.

### Computation

To model EGFR signaling network, we adopted a deterministic ordinary differential equation (ODE) model. Model scheme is described in Additional file 1, Table S1-5. Additional file 1, Table S1 and 2 summarize the biochemical reactions with 29 components and 27 differential equations, which are given by mass action or Michaelis-Menten kinetics. Additional file 1, Table S3 and 4 list the parameter values and the initial concentrations of the cellular signaling molecules. These values were estimated based on the parameter ranges which were listed in Additional file 1, Table S5. Our pathway network is drawn by Cell Designer 4.1 which is an Systems Biology Markup Language (SBML)- compliant application, and is available with this publication (see Additional file 2).

The parameter estimation problem is defined as a function optimization problem to minimize the sum of the squared error:

$$ERR=\sum_{\begin{array}{l} \text{Cell Type}\\ \in \text{\{WT, EGFR-WT, L858R models\}} \end{array}}\sum_{\begin{array}{l} \text{Protein}\\ \in P \end{array}}\sum_{\begin{array}{l} \text{DataPoint}\\ \in D \end{array}}{\left\{\text{Experimental data}–\text{Simulated value}\right\}}^{\text{2}},$$

where the sets *P *= {phosphorylated EGFR, Shc, MEK, ERK} and *D *= {1, 5, 10, 30 minutes after EGF stimulation} are an experimentally observed values (Additional file 1, Table S6). We used a technique to decompose the parameter estimation problem of our model into two subproblems, reaction steps from 1 to 17 (A) and from 18 to 27 (B). The problem decomposition technique is an effective means to resolve the high-dimensionality and can only be applied to enzymatic reactions given by Michaelis-Menten kinetics. Since the upstream region affect the downstream subproblem, the subproblems were solved in order, from the upstream (A) to the downstream (B). As a parameter estimator, we used the genetic algorithm with Genetic Local Search with distance independent Diversity Control (GLSDC) by extending the basic idea of a genetic algorithm with Distance Independent Diversity Control (DIDC) to coarse grained parallelization [38]. The GLSDC program was executed on the the RIKEN Integrated Cluster of Clusters (RICC) system.

The model was implemented with MATLAB R2008a (The Mathworks, Inc.), and ''ode15s'' function was applied to solve the ODEs (http://www.mathworks.com/access/helpdesk/help/techdoc/ref/ode113.html).

"ode15s" function is a variable order solver based on the numerical differentiation formulas (NDFs) and is a multistep solver. The function is used when the problem is a differential-algebraic or stiff equation (http://www.mathworks.com/access/helpdesk/help/techdoc/ref/ode23.html#f92-998740).

### Additive, antagonistic, or synergistic effect classification analysis

Classification of additive, antagonistic, or synergistic effect is determined by comparing the response to a combinatorial perturbation with that to a single perturbation [39,40]. This classification analysis has been mainly used to categorize the efficacy of combinatorial drugs into three types, considering drug dose as a perturbation. In the present analysis, we made some modifications not to lose the original meaning so that we could categorize the ERKPP inhibitory effect by the combinatorial perturbations of Mig6 effect and gefitinib. As an index for the efficacy of perturbation, we used the concentration of ERKPP at *t *= 5 minutes. The perturbation for the administration of gefitinib was expressed by varying the parameter *k*_3 _(the rate constant for the forward reaction of EGFR phosphorylation). The perturbation for the effect of Mig6 was expressed by varying both *k*_3 _(the rate constant for the forward reaction of EGFR phosphorylation) and *k*_8 _(the rate constant for the forward reaction of binding of EGFR to Shc), because only these two parameters contribute to the strong inhibition of ERKPP, although we assumed that four parameters (*k*_3_*, k*_5_*, k*_7_, and *k*_8_) were affected by Mig6 in L858R model A. Given the values of *k*_3 _or *k*_8 _that individually achieve *X*/2% ERKPP inhibition, the value of paired perturbation additively produces *X*% ERKPP inhibition. Therefore, the combinatorial effect at an inhibitory intensity *X *is categorized as additive, antagonistic, or synergistic according to whether the paired perturbation produces ERKPP inhibition equal to, less than, or more than *X*%. For example, the combination of the value of *k*_3 _and *k*_8 _which result in 10% inhibition of ERKPP individually is categorized into additive, antagonistic, or synergistic effect, according to whether the inhibition level in ERKPP is equal to, less than, or more than 20%.

## Results

### Mathematical model of EGFR-ERK signaling in H1299 lung cancer cells

**Figure 1:**
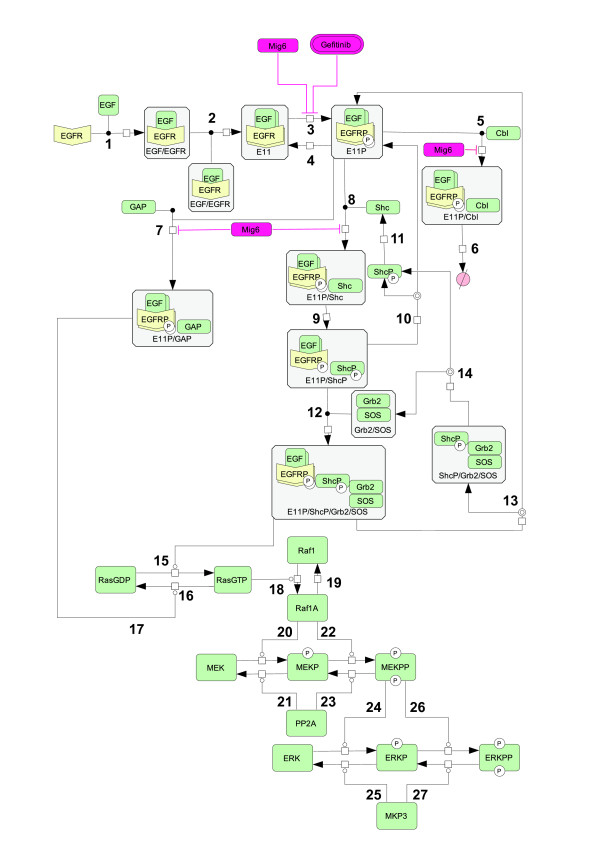
**Reaction scheme for EGFR signal transduction pathway in H1299 cells**. The reaction steps of the EGFR signaling pathway are labeled from 1 to 27. These numbers correspond to the biochemical reactions in Additional file 1, Table S1. Steps 4, 11, and 15-27 are described by Michaelis-Menten kinetics and the other reactions by mass action. The individual reaction equations, the estimated parameters, and the estimated ranges are explained in Additional file 1, Table S2-5. The inhibitory effects of Mig6 and gefitinib are indicated by red lines. E11: EGFR homodimer; ShcP: free phosphorylated Shc; E11P: phosphorylated EGFR homodimer; Raf1A: activated Raf1; MEKP: singly phosphorylated MEK; MEKPP: doubly phosphorylated MEK; ERKP: singly phosphorylated ERK; ERKPP: doubly phosphorylated ERK.

To evaluate the dynamics of the signal transmission from EGFR to its downstream elements, we used the network model introduced by Kholodenko *et al *[25], Hatakeyama *et al. *[28], and Wolf *et al *[34] with some modifications. Figure 1 shows the EGFR signaling pathway considered in the current study. The pathway starts from the EGFR located in the cell membrane and is composed of 27 reaction steps. EGF first binds to the EGFR and causes receptor dimerization, then receptor autophosphorylation occurs at particular tyrosine residues in the cytoplasmic domains (steps 1-3) [41-43]. Phosphorylated EGFR is dephosphorylated by protein phosphotyrosine phosphatases (step 4) [44,45]. Some of the phosphorylated dimers are internalized by binding of Cbl and subsequently degraded (steps 5 and 6) [46,47]. This receptor degradation is one of the most important processes for preventing over-signaling. Other phophorylated dimers associate with the Grb2 (growth factor receptor-bound protein 2)/SOS (son of sevenless) complex via Shc (Src homology and collagen domain protein) (steps 8, 9, and 12) [48-50]. This complex can dissociate, yielding the EGFR dimer, the Grb2/SOS complex, and Shc (steps 10, 11, 13, and 14). After recruiting Grb2/SOS with the phosphorylated EGFR dimer to the plasma membrane, SOS activates Ras by exchanging GDP for GTP (step 15) [51,52]. In an opposing reaction, deactivation of Ras is accelerated by GAPs (GTPase-activating proteins) associated with EGFR (steps 7, 16, and 17). Binding of GAP to the phosphorylated EGFR is a key step to control the output of the signaling pathway [53-55]. EGF stimulation induces recruitment of GAP to the membrane [31], and GAP is strongly activated after binding to the phosphorylated EGFR. Ras deactivation normally keeps its GTPase activity low. The GTP-bound Ras can then translocates Raf1 to the cell membrane for its activation (step 18) [56,57], which is also reversible (step 19). Activated Raf1 activates MEK by phosphorylation of two serine residues (steps 20 and 22), and the activated MEK phosphorylates ERK on threonine and tyrosine residues (steps 24 and 26) [58,59]. The MAPK cascade is negatively regulated by PP2A (protein phosphatase 2A) for the dephosphorylation of MEK (steps 21 and 23) and by MKP3 (MAPK phosphatase 3) for the dephosphorylation of ERK (steps 25 and 27) [60,61]. After its translocation to the nucleus, activated ERK regulates gene expression by phosphorylating transcription factors such as Elk and Myc [58,62-65].

To investigate cell-specific EGFR signaling dynamics, we constructed an H1299WT model (WT model), an H1299EGFR-WT model (EGFR-WT model), and two alternate H1299L858R models (L858R model A with Mig6 overexpression and L858R model B with Cbl overexpression). The differences among these models are summarized in Table 1. To simulate the effect of EGFR overexpression in H1299EGFR-WT and H1299L858R cells, the initial concentrations of EGFR in the respective models were assumed to be higher than that in the WT model. Also, we constructed two L858R models based on H1299L858R cell-specific characteristics. In the first model (L858R model A), Mig6 was added to the EGFR-WT model because Mig6 is highly endogenously expressed in H1299L858R cells [18]. For simplicity, the effect of Mig6 was not described explicitly, but was realized by modifying four parameters in the EGFR-WT model. The modified parameters were the rate constant for the forward reaction of the EGFR phosphorylation (*k*_3_), the binding of EGFR to Cbl (*k*_5_), GAP (*k*_7_), and Shc (*k*_8_) (steps 3, 5, 7, and 8). These modifications were included to mimic the effect of Mig6 overexpression, which leads to suppression of EGFR phosphorylation and binding of the EGFR dimer to other proteins [19,21]. In the second model (L858R model B), the initial concentration of Cbl was increased compared to that in the EGFR-WT model, because H1299L858R cells showed an increase in ubiquitination compared to H1299EGFR-WT cells [15] and receptors in Cbl overexpressing cells underwent more rapid ligand-induced ubiquitination compared to control cells [66]. The values of other parameters were the same as those in the EGFR-WT model.

**Table 1:** Differences in characteristics among the four H1299 models.

	Circumstances	Model description
**WT model**	-----	-----

**EGFR-WT model**	EGFR overexpression	Increased EGFR initial concentration

	EGFR overexpression	Increased EGFR initial concentration
	
		Slow rate for the forward reaction of EGFR phosphorylation (*k*_3_)
**L858R model A**	Mig6 overexpression	Slow rate for the forward reaction of binding of phosphorylated EGFR to Cbl (*k*_5_)
		Slow rate for the forward reaction of binding of phosphorylated EGFR to GAP (*k*_7_)
		Slow rate for the forward reaction of binding of phosphorylated EGFR to Shc (*k*_8_)

	EGFR overexpression	Increased EGFR initial concentration
	
**L858R model B**	Increased ubiquitin-dependent EGFR degradation	Increased Cbl initial concentration

(comparison with WT model)

### Time-course and dose-dependent phosphorylation by EGF stimulation

**Figure 2:**
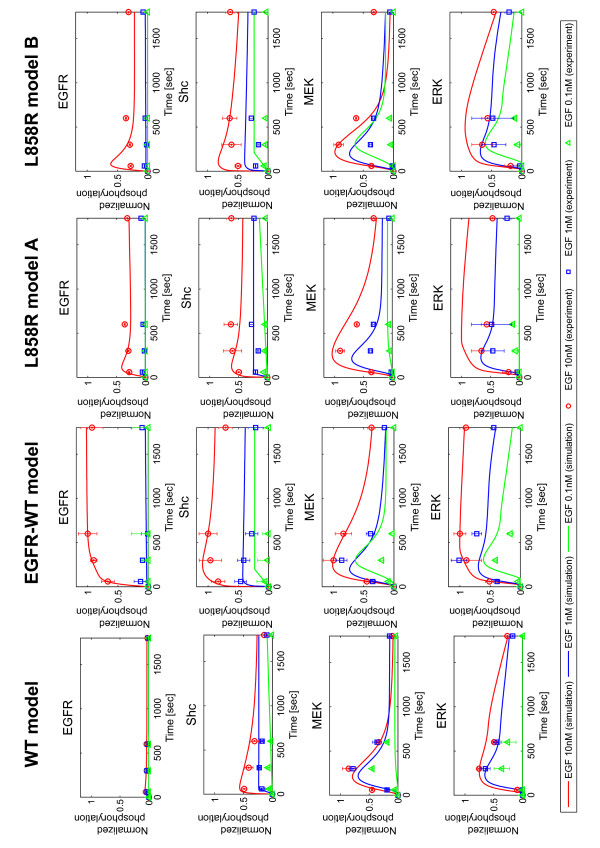
**Time-course of the EGF-induced phosphorylation in the H1299 cells**. Phosphorylation patterns of EGFR, Shc, MEK, and ERK in H1299WT (first panel from left), H1299EGFR-WT (second panel from left) and H1299L858R (third and forth panel from left) cell lines. Experimental results are shown as symbol plots. Western blot experiment was repeated twice, and mean values of the signal intensities were calculated. The values were normalized for each protein so that the maximum values were equal to 1 and the values at *t *= 0 minutes were equal to 0 under the assumption that all the proteins were inactive before EGF stimulation. Cells were treated with three concentrations of EGF: 10 nM (red circle), 1 nM (blue square), and 0.1 nM (green triangle). Representative western blot images are shown in Additional file 1, Figure S1A. Simulation results are shown as solid lines. The lines represent the time-course phosphorylation levels *in silico*. Red, blue, and green lines correspond to simulation results with three concentrations of EGF: 10 nM, 1 nM, and 0.1 nM, respectively.

First, we used experimental and computational approaches to investigate the time-course of EGFR signaling dynamics. Figure 2 and Additional file 1, Figure S1A show the experimental results. The graphs show the time-courses of phosphorylation levels after stimulation with 0.1, 1, and 10 nM EGF measured for four key proteins: EGFR, Shc, MEK and ERK. H1299EGFR-WT cells showed the highest level of phosphorylation in all four proteins, whereas the H1299WT cells showed the lowest and the H1299L858R cells were intermediate. Overexpression of EGFR induced sustained and strong signaling activity, while the L858R mutation reduced signaling particularly in the upstream part of the signaling pathway (EGFR and Shc). In contrast, the differences in the time-course kinetics among the three derivatives became less obvious in the downstream part of the signaling pathway (MEK and ERK). The simulation results of WT, EGFR-WT, and two L858R models for EGF stimulation (0.1, 1 and 10 nM) are shown in Figure 2 and Additional file 1, Figure S2. As described in the previous section, most of the parameters were common among the four models. Despite using these common parameters, the models successfully captured the variations in time-course activation dynamics, and the simulation results were fairly consistent with the experimental results for all three H1299 cell derivatives (Figure 2).

**Figure 3:**
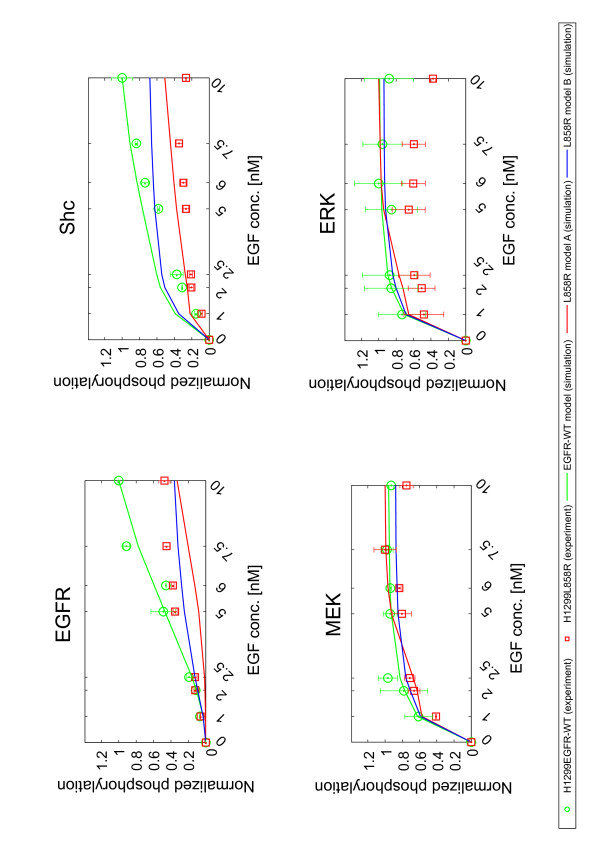
**EGF-dose dependent phosphorylation of signaling molecules**. Phosphorylation of EGFR, Shc, MEK, and ERK in H1299EGFR-WT (green circle) and H1299L858R (red square) cells at 5 minutes after administration of the indicated concentrations of EGF. Western blots were performed twice for each protein, and the mean values are displayed together with standard deviations. Representative western blot images are shown in Additional file 1, Figure S1B. Experimental results are shown as symbol plots. Simulation results are shown as solid lines. The values were normalized for each protein so that the maximum values were equal to 1.

Next, the EGF ligand-dose response was examined to investigate how the L858R mutation affects cooperativity of EGFR signaling. Figure 3 and Additional file 1, Figure S1B show the experimental results in the phosphorylation levels of EGFR, Shc, MEK, and ERK in the EGFR pathway when the dose of EGF was varied. The phosphorylation levels of EGFR and Shc gradually increased as a function of the EGF concentration in both H1299EGFR-WT and H1299L858R cells, while MEK and ERK were highly phosphorylated even at EGF concentrations as low as 1 nM. The phosphorylation of EGFR and Shc in the H1299L858R cells showed a smaller dynamic range compared to that of the H1299EGFR-WT cells, whereas there was no significant difference between the two cell types in MEK and ERK phosphorylation patterns. Again, the L858R mutation reduced the phosphorylation levels in the upstream part of the signaling pathway (EGFR and Shc) in an EGF-dose dependent manner, but had little effect on the downstream part (MEK and ERK). The simulation results imply that the models fairly reproduced the ligand-dose dependent behavior of the H1299EGFR-WT and H1299L858R cells (Figure 3). Also, both L858R models A and B seem to equally reproduce the time-course and the EGF dose-dependency obtained with the H1299L858R cells. Additional analyses are required to distinguish the model that represents the behaviors of the signaling dynamics associated with L858R mutation.

### Mig6 plays a role in transmitting the effect of gefitinib to the downstream part of the EGFR signaling pathway

**Figure 4:**
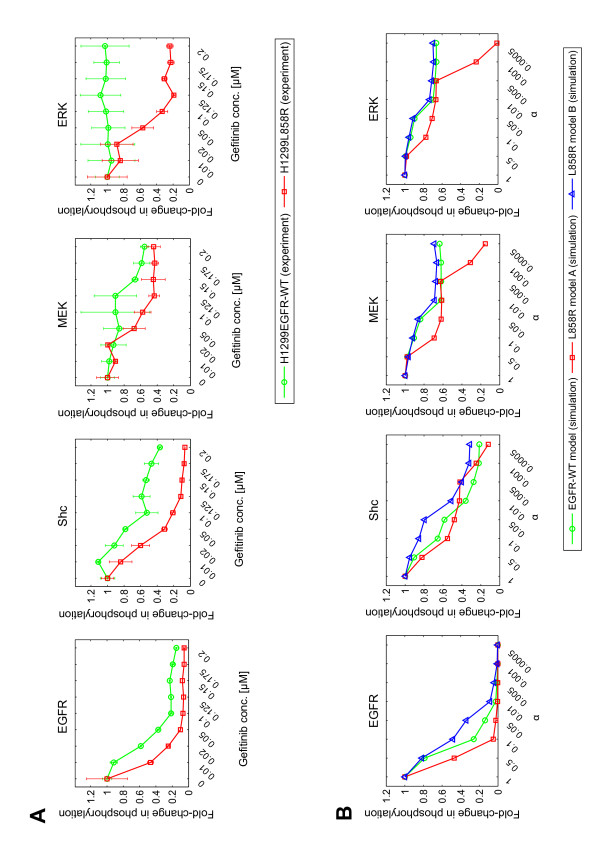
**Gefitinib-dose dependent effects on the phosphorylation of signaling molecules**. **(A) **Experimental results. Phosphorylation of EGFR, Shc, MEK, and ERK in H1299EGFR-WT (green circle) and H1299L858R (red square) cells was measured after 5 minutes of EGF (10 nM) administration, in the presence of gefitinib at the indicated concentrations. Western blots were performed twice, and the mean values and the standard deviations of the signal intensities were calculated. The values were normalized for each protein so that the values without gefitinib were equal to 1. Representative western blot images are shown in Additional file 1, Figure S1C. **(B) **Simulation results. The gefitinib effect was simulated by multiplying the kinetic parameter of *k*_3 _(the rate constant for the forward reaction of EGFR phosphorylation). The multiplying coefficient *α *is given by *α *= (*k*_3_)/(*k*_3 _in EGFR-WT model or L858R models). The green circles (EGFR-WT model), red squares (L858R model A), and blue triangles (L858R model B) show concentrations of phosphorylated EGFR, Shc, MEK, and ERK at *t *= 5 minutes, plotted with several values of *α *(1, 0.5, 0.1, 0.05, 0.01, 0.005, 0.001 and 0.0005). *α *= 1 means without gefitinib.

To clarify unknown other factors that affect gefitinib sensitivity, we investigated the gefitinib-dose response effect on the EGFR signaling pathway. Figure 4A and Additional file 1, Figure S1C show the experimental results of phosphorylation of the signaling proteins in the presence of different doses of gefitinib. The H1299EGFR-WT and H1299L858R cells showed similar dose-dependent responses in the upstream proteins EGFR and Shc. However, the H1299L858R cells were more sensitive to gefitinib administration at the phosphorylation of the downstream proteins, MEK and especially ERK, compared to the H1299EGFR-WT cells, which were essentially insensitive to gefitinib for ERK phosphorylation (Figure 4A). Figure 4B shows the simulation results of the gefitinib dose-response. The effect of the gefitinib was mimiced by changing the kinetic parameter *k*_3 _of EGFR phosphorylation. *α *indicates the multiplying coefficient for *k*_3_. The results indicate that the L858R model A, including Mig6, successfully reproduced the inhibitory effect for ERK at higher concentrations of gefitinib, whereas model B, which included the effect of Cbl, failed to reproduce this response. We confirmed that L858R model A with other parameter sets that have similar cost function values yields the same trends with regard to the role of Mig6 in gefitinib sensitivity (data not shown). Also, we found that EGFR-WT model at lower concentrations of EGF is as sensitive to gefitinib for ERK phosphorylation as L858R model A (data not shown). Based on these results, model B is inappropriate as the mechanism to explain the gefitinib sensitivity, whereas model A remains as a viable candidate.

**Figure 5:**
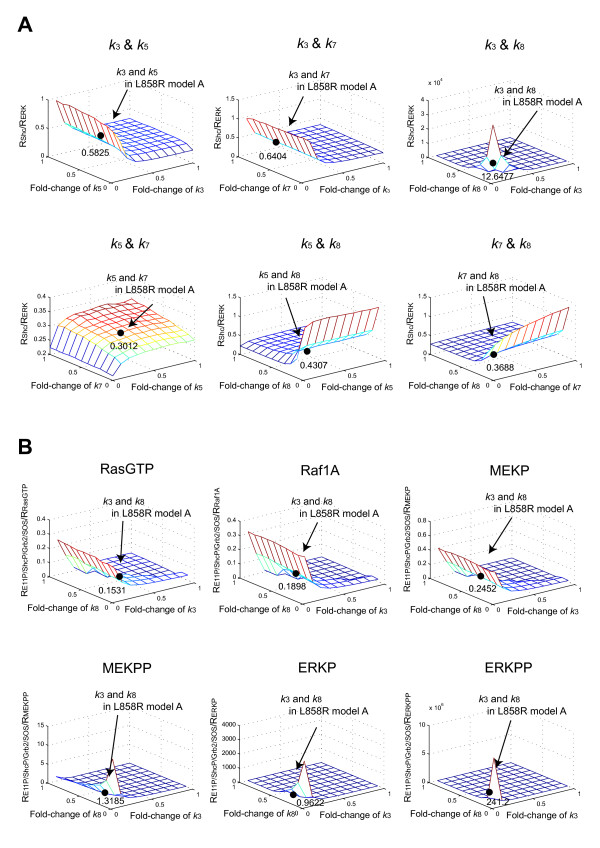
**Mechanism for enhancing gefitinib sensitivity of the downstream signaling steps**. **(A) **Ratios of phosphorylated Shc to phosphorylated ERK in the presence of gefitinib with varying Mig6 effect parameters (*k*_3_, *k*_5_, *k*_7_, and *k*_8_). Total phosphorylated Shc and ERK measured at 5 minutes after EGF (10 nM) stimulation were used as the upstream output and as the downstream output, respectively. *R*_X _was defined as *R*_X _= [total phosphorylated X at *α *= 0.0005]/[total phosphorylated X at *α *= 1]. The values of *R*_Shc_/*R*_ERK _were calculated for each combination of two parameters selected from four Mig6 effect parameters (six combinations such as *k*_3 _&*k*_5_, *k*_3 _&*k*_7_, *k*_3 _&*k*_8_, *k*_5 _&*k*_7_, *k*_5 _&*k*_8_, and *k*_7 _&*k*_8_). In the case of *k*_3 _&*k*_5 _combination, for example, the simulation at *k*_3 _= 1 and *k*_5 _= 1 shows EGFR-WT model and the value in the *k*_3 _&*k*_5 _panel indicate *R*_Shc_/*R*_ERK _for *k*_3 _and *k*_5 _in L858R model A and *k*_7 _and *k*_8 _in EGFR-WT model. The other combinations are given in the similar manner. **(B) **Ratios of E11P/ShcP/Grb2/SOS to RasGTP, Raf1A, MEKP, MEKPP, ERKP, and ERKPP with varying *k*_3 _and *k*_8_. E11P/ShcP/Grb2/SOS measured at 5 minutes after EGF (10 nM) stimulation was used as the upstream output. RasGTP, Raf1A, MEKP, MEKPP, ERKP, and ERKPP measured at 5 minutes after EGF (10 nM) stimulation were used as the downstream output. *R*_Y _is defined as *R*_Y _= [Y at *α *= 0.0005]/[Y at *α *= 1]. The values of *R*_E11P/ShcP/Grb2/SOS_/*R*_Y_, were calculated by using various values of *k*_3 _and *k*_8 _(Y: RasGTP, Raf1A, MEKP, MEKPP, ERKP, and ERKPP). In the case of RasGTP, for example, the simulation at *k*_3 _= 1 and *k*_8 _= 1 shows EGFR-WT model and the value in the RasGTP panel indicate *R*_E11P/ShcP/Grb2/SOS_/*R*_RasGTP _for *k*_3 _and *k*_8 _in L858R model A and *k*_5 _and *k*_7 _in EGFR-WT model. The other panels are given in the similar manner.

Although the sensitivity to inhibition of phosphorylation by gefitinib was different in the downstream proteins (MEK and ERK) between H1299EGFR-WT and H1299L858R cells, the difference was small in the upstream proteins (EGFR and Shc). Therefore, it seemed likely that particular reaction steps in the pathway would amplify the small difference observed in the upstream proteins (EGFR and Shc). To identify the critical steps, we analyzed the contribution of each parameter to the downstream phosphorylation influenced by gefitinib. We used an index that indicates the ratios of the upstream signaling activity to the downstream signaling activity in the presence of gefitinib. The output of the upstream signaling was the total phosphorylated Shc, and the downstream output was the total phosphorylated ERK. When *R*_X _is defined as the ratios of total phosphorylated X with gefitinib to total phosphorylated X without gefitinib, the index *R*_Shc_/*R*_ERK _was calculated for every combination of the four parameters (*k*_3_, *k*_5_, *k*_7_, and *k*_8_) affected by Mig6. As a result, the combination of a lower *k*_3 _(the rate constant for the forward reaction of the EGFR phosphorylation) and *k*_8 _(the rate constant for the forward reaction of binding of EGFR to Shc) contributed to effectively inhibit the total phosphorylation of ERK in the presence of gefitinib (Figure 5A). The values in each panel indicate *R*_Shc_/*R*_ERK _calculated by using the values of parameters in L858R model A for two changing parameters and those in EGFR-WT model for two unchanging parameters. The value of *R*_Shc_/*R*_ERK _for L858R model A is 10.6520. Next, we analyzed which downstream reactions were influenced by the combination of these two parameters. In this case, E11P/ShcP/Grb2/SOS was used as the upstream output, and the downstream outputs were RasGTP, Raf1A, MEKP, MEKPP, ERKP, and ERKPP. When *R*_Y _is defined as the ratios of Y with gefitinib to Y without gefitinib, Figure 5B shows *R*_E11P/ShcP/Grb2/SOS_/*R*_Y _by varying the values of *k*_3 _and *k*_8 _(Y: RasGTP, Raf1A, MEKP, MEKPP, ERKP, and ERKPP). The values in each panel indicate *R*_E11P/ShcP/Grb2/SOS_/*R*_Y _calculated by using the values of *k*_3 _and *k*_8 _in L858R model A and *k*_5 _and *k*_7 _in EGFR-WT model. Based on this analysis, we found high gefitinib sensitivity in MEKPP, ERKP, and ERKPP. These results indicate that the steps of MEK phosphorylation/dephosphorylation (steps 22 and 23) and ERK phosphorylation/dephosphorylation (steps 24-27) amplify the gefitinib sensitivity.

### The combination of Mig6 and gefitinib has a synergistic effect in inhibiting EGFR signaling

**Figure 6:**
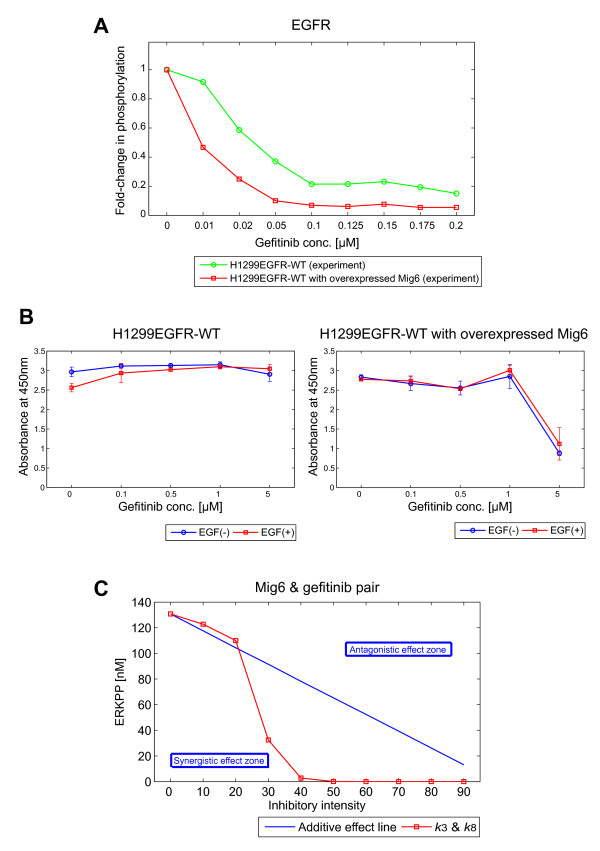
**Mig6 overexpression synergistically enhances the cell growth inhibitory effect of gefitinib**. **(A) **EGFR phosphorylation influenced by Mig6 overexpression in the presence of gefitinib. Phosphorylation of EGFR in H1299EGFR-WT cells (green circle) and H1299EGFR-WT cells with overexpressed Mig6 (red square) was measured 5 minutes after EGF (10 nM) administration, in the presence of gefitinib at the indicated concentrations. Western blots were performed twice, and the mean values of the signal intensities were calculated. The mean values were normalized so that the values without gefitinib were equal to 1. **(B) **Cell growth influenced by Mig6 overexpression in the presence of gefitinib. Cell viabilities of H1299EGFR-WT cells (left panel) and H1299EGFR-WT cells with overexpressed Mig6 (right panel) were measured by an MTT assay 3 days after stimulation without (blue circle) or with (red square) 10 nM EGF in the presence of various doses of gefitinib (0, 0.1, 0.5, 1, and 5 μM). The data shown are the means and the standard deviations of triplicated or quadruplicated experiments. **(C) **Inhibitory effect of a combination of Mig6 and gefitinib (Mig6 & gefitinib pair). Red squares show concentrations of ERKPP at *t *= 5 minutes under paired perturbations *in silico*, plotted against inhibitory intensities *X *(0, 10, 20, 30, 40, 50, 60, 70, 80, and 90). See Materials and Methods section for the explanation of *X*. The blue line shows the effect expected from a linear interpolation of two individual perturbations (additive effect line). The area above the additive line shows a smaller effect than the additive effect (antagonistic effect zone), and the area below it shows a stronger than additive effect (synergistic effect zone).

The only difference between the EGFR-WT model and the L858R model A is in the negative EGFR regulation produced by Mig6, therefore, which can be experimentally verified by overexpressing Mig6. We have known that expression level of Mig6 is reversely correlated with ERK phosphorylation level in the H1299 derivatives with various EGFR mutations [18]. To study its effect on the upstream signaling, we performed western blotting for phosphorylated EGFR. Figure 6A shows that Mig6 overexpression more inhibited EGFR phosphorylation in the presence of gefitinib. Therefore the effect of Mig6 for gefitinib administration was further studied using MTT cell proliferation assay. At a high concentration (5 μM) of gefitinib, cell growth was suppressed in the cells with Mig6 overexpression, but not in the H1299EGFR-WT cells (Figure 6B). This result indicates that Mig6 indeed enhances the inhibitory effect of gefitinib as our mathematical model had predicted.

We next analyzed the effects of a combinatorial perturbation of Mig6 and gefitinib on the signaling inhibition. Combinations of perturbations can be categorized into three interaction types: additive, antagonistic, or synergistic, according to whether the combination of two perturbations produces an effect equal to, less than, or larger than that expected based on the individual effects of the single perturbations [39,40]. We analyzed the effect of the combination of Mig6 and gefitinib on ERKPP (see Material and Methods section for a detailed description of the analysis method) and found that the combination of Mig6 and gefitinib exhibits a synergistic effect (Figure 6C). This result indicates that synergism is produced by the dual inhibition of the step of EGFR phosphorylation (*k*_3_) which both Mig6 and gefitinib inhibit and the step of binding of EGFR to Shc (*k*_8_) which Mig6 alone inhibits.

## Discussion

Overexpression or mutation of EGFR has been observed in lung cancers [9,67,68], and these molecular changes affect the prognosis and treatment sensitivity of patients [10,69-74]. Those abnormalities could cause changes in overall titers of signaling networks at the molecular, the cellular, and even the individual levels. Mathematical model is helpful for understanding of the mechanical aspects of interconnected signaling network and predicting input-output behaviors in the pathway. However, it is often very difficult to explain the variation of reactants in signal transduction pathway using a single unified mathematical model. In this paper, we attempted to build such a unified model that could uncover the cell-specific regulatory mechanisms produced by overexpression and mutation of the EGFR and the association with gefitinib sensitivity.

The model used in this paper successfully reproduced the experimental observations concerning the activation of the key proteins in the pathway and discriminated the roles of Mig6 and Cbl in gefitinib sensitivity. The model was based on kinetic equations, and most of the parameters for these equations were common for all models. The differences in the parameters were confined to specific steps and proteins - EGFR overexpression, inhibitory effects caused by Mig6, and Cbl overexpression leading to the degradation of EGFR.

Our results revealed that the effectiveness of gefitinib in cells is largely affected by not only on its direct binding affinity with EGFR but also on the presence of an additional molecule, Mig6. According to recent reports, the sensitivity to kinase inhibition reflects intrinsic differences in the binding affinity of the EGFR mutants such as L858R, G719S, and exon19 deletions [16,75-78]. Yun *et al *[16] showed that gefitinib directly binds more tightly to the L858R mutant than to the wild type EGFR *in vitro*, while Fabian *et al *[17] indicated that EGFR with gefitinib sensitive mutations does not differ from wild type EGFR in terms of gefitinib binding affinity. This would suggest that the stronger interaction of the mutated EGFRs with gefitinib may not be the only one mechanism for the good clinical response to gefitinib in NSCLC [17]. This inconsistency in previous reports suggested to us that unknown factors may play an important role in gefitinib sensitivity. The gefitinib-dose response study (Figure 4) showed that the difference in the phosphorylation levels of the downstream proteins (MEK and ERK) inhibited by gefitinib is large between H1299EGFR-WT and H1299L858R cells, whereas the difference in the upstream proteins (EGFR and Shc) is small. By a detailed computational analysis, we found that Mig6 has an important role in propagating the gefitinib effect at the steps of MEK phosphorylation/dephosphorylation and ERK phosphorylation/dephosphorylation. Additionally, the combination of the inhibition of EGFR phosphorylation (the effect of Mig6 and gefitinib) and the inhibition of binding of EGFR to Shc (the effect of Mig6) produced a synergistic inhibitory effect on EGFR signaling. Therefore, Mig6 could be one of the critical factors to explain gefitinib sensitivity at cellular level. We constructed the model by referring to the earlier studies on Mig6 functions [20-22]. However, the model could be modified and improved when novel mechanism of Mig6 in the regulation of EGFR or new regulators associated with the EGFR L858R mutation are identified by further studies.

Our results shown in Figures 2 and 3 are consistent with a previously published report that EGFR with the L858R mutation did not have stronger EGFR phosphorylation upon EGF stimulation when compared to the wild type EGFR in H1299 cells [15]. On the other hand, Guha *et al *[79] and Yun *et al *[16] observed significantly high phosphorylation of the L858R-mutated EGFR compared with wild type EGFR expressed in HBEC cells and Sf9 cells. This inconsistency among cell types may be explained by the relative levels of Mig6, which is highly expressed when EGFR kinase is in an active state.

## Conclusion

Overall, the analysis presented in this paper allows understanding of the impacts of cancer-related abnormalities on the EGFR signaling pathway. Also, we demonstrate the feasibility of using computational models to predict one of the determinants for the evaluation of drug sensitivities. Despite the fact that a new drug may help prevent the deaths of thousands of patients, there are many instances where the patients become severely ill or die because of serious unwanted side-effects. Hence, in prescribing medications appropriate for individual patients, there is a clear need for guidance in predicting side-effects and drug sensitivity. It would be no exaggeration to say that the side-effects could not be predicted in advance, since signaling pathways are very complex. We believe that in part such guidance can be predicted by computational modeling of appropriate signaling pathways.

## Authors' contributions

YN carried out the mathematical modeling and analysis work, and wrote the manuscript. TNag helped to draft the manuscript. RU, YS, and SFT carried out the experimental laboratory work. TNakak and TNaka proposed a basic idea of the mathematical methods. HT participated in the design and coordination of the study. All of the work was supervised by MO. All authors have read and approved the final version of the manuscript.

## Supplementary Material

Additional file 1 **Supplementary Information**. This PDF file contains all additional figures and tables referenced in the text. Click here for file

Additional file 2 **H1299 model**. This XML file contains our model represented by SBML. Click here for file
