# Supplementary material for: Epidermal growth factor receptor mutation in combination with expression of MIG6 alters gefitinib sensitivity
Source: BMC Syst Biol. 2011 Feb 18;5:29. doi: 10.1186/1752-0509-5-29 (PMC3224393; doi:10.1186/1752-0509-5-29)
Supplement: Additional file 1 — Supplementary Information. This PDF file contains all additional figures and tables referenced in the text. [file 1752-0509-5-29-S1.PDF]

SUPPLEMENTARY INFORMATION

Epidermal growth factor receptor mutation in combination with expression of MIG6 alters gefitinib sensitivity

Yoshimi Naruo, Takeshi Nagashima, Ryoko Ushikoshi-Nakayama, Yuko Saeki, Takashi Nakakuki, Takashi Naka, Hiroshi Tanaka, Shih-Feng Tsai and Mariko Okada-Hatakeyama

Contents:

Supplementary Figure 1

Representative western blot figures showing phosphorylation of EGFR, Shc, MEK, and ERK in H1299 derivatives cell lines.

Supplementary Figure 2

Computer simulation of 29 reactants for EGFR signal transduction in the four H1299 models.

Supplementary Table 1

Biochemical reactions for the H1299 model.

Supplementary Table 2

Ordinary differential equations for the model.

Supplementary Table 3

Estimated kinetic constants for the H1299 models.

Supplementary Table 4

Estimated initial concentrations of the cellular components.

Supplementary Table 5

Search range for parameter estimation.

Supplementary Table 6

Dataset for parameter estimation.

SUPPLEMENTARY FIGURES

Supplementary Figure 1. Representative western blot figures showing phosphorylation of EGFR, Shc, MEK, and ERK in H1299 derivatives cell lines.

(A) EGF-induced time-course phosphorylation profiles of EGFR, Shc, MEK and ERK in H1299WT, H1299EGFR-WT and H1299L858R cells. **(B)** EGF-dose dependent phosphorylation in H1299EGFR-WT and H1299L858R cells. **(C)** Dose-dependent effect of gefitinib for EGF (10 nM)-stimulated phosphorylation. For each experiment, western blot analysis was performed independently twice. Quantified signal intensities of phosphorylated proteins in (A), (B), and (C) were normalized for each protein and are shown in Figure 2A, 3A, and 5A, respectively.

Supplementary Figure 2. Computer simulation of 29 reactants for EGFR signal transduction in the four H1299 models.

Red, blue, and green lines correspond to simulation results with three concentrations of EGF: 10 nM, 1 nM, and 0.1 nM, respectively. **(A)** WT model; **(B)** EGFR-WT model; **(C)** L858R model A; **(D)** L858R model B.

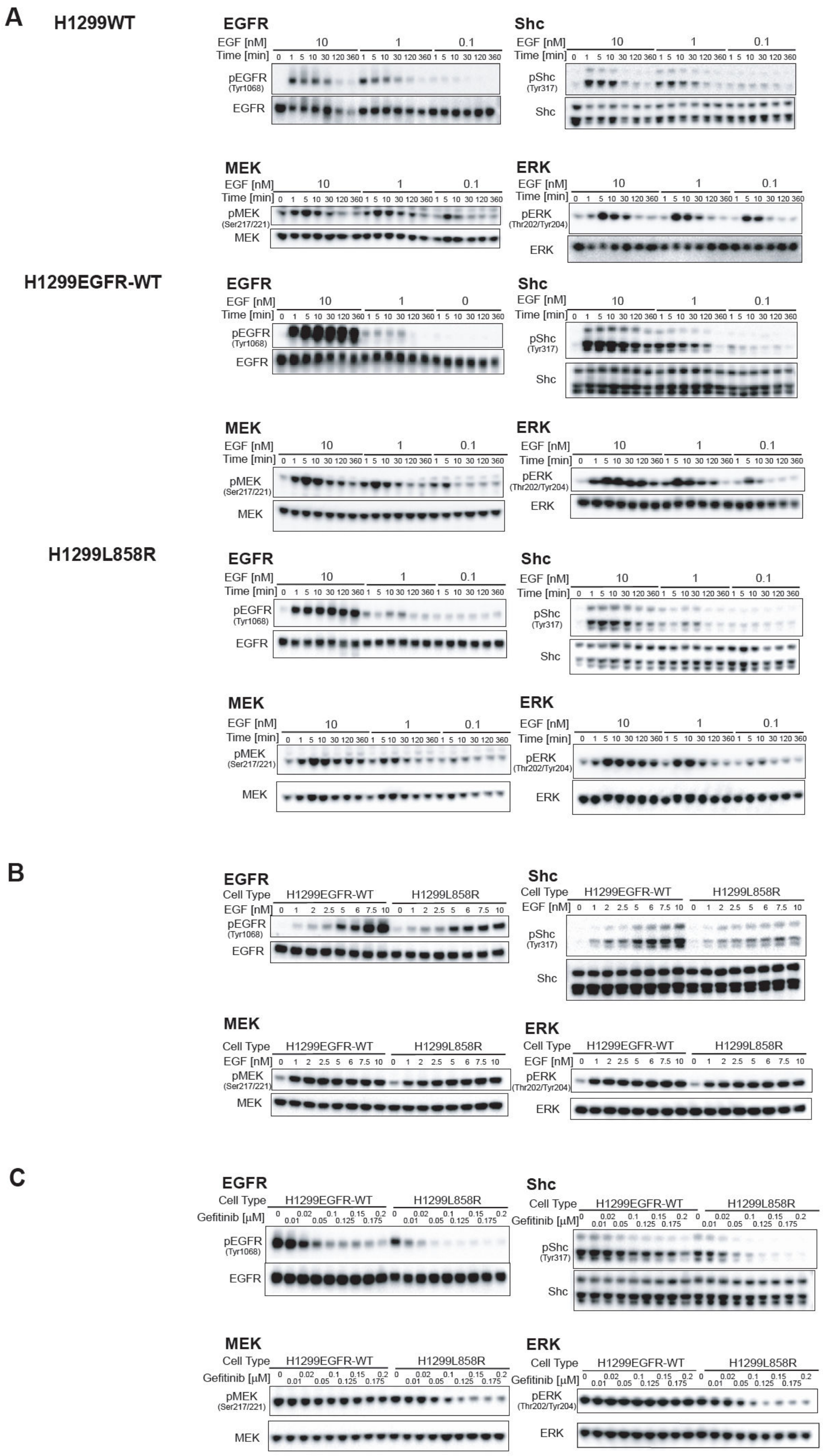

Supplementary Figure 1

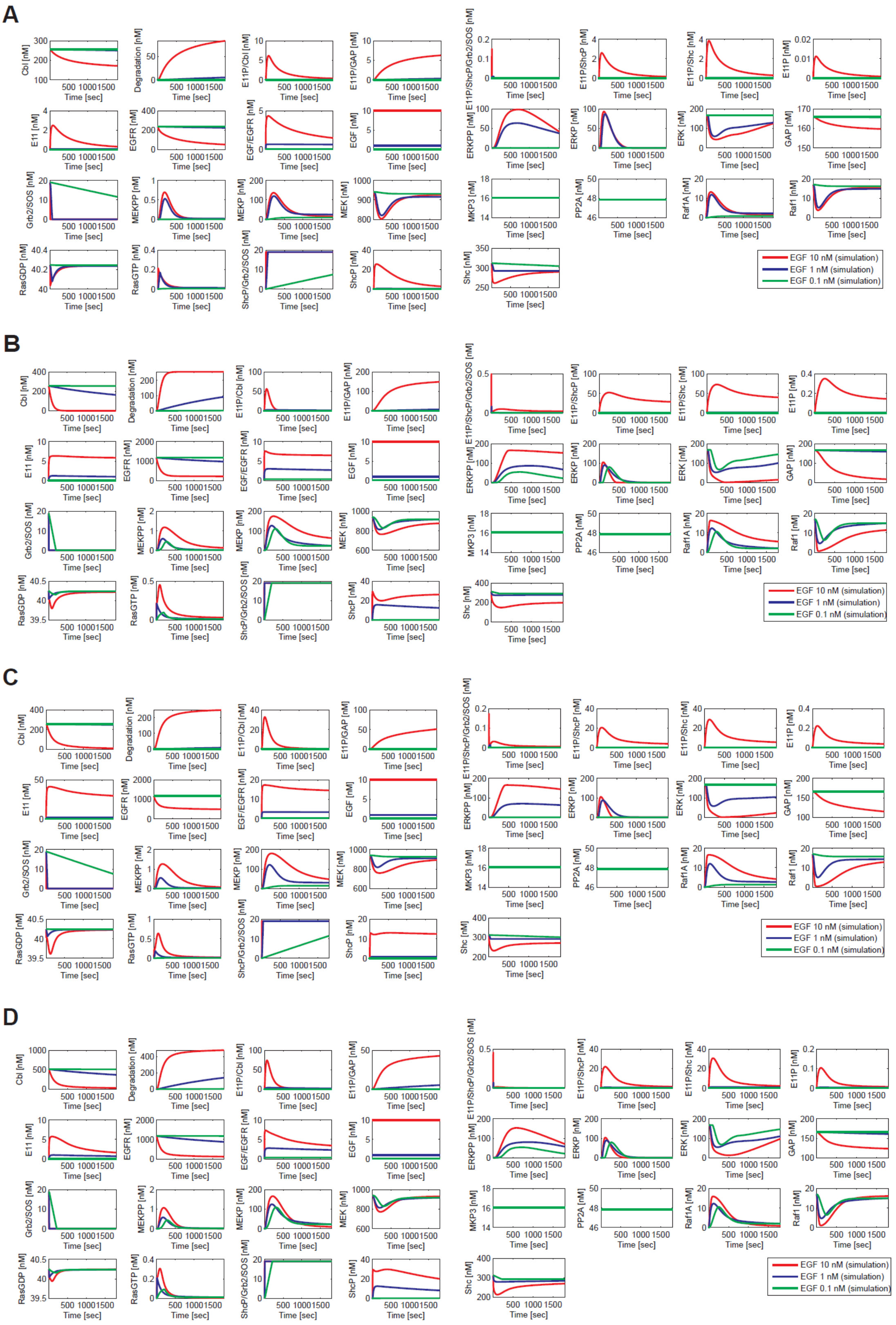

Supplementary Figure 2

SUPPLEMENTARY TABLES

Supplementary Table 1. Biochemical reactions for the H1299 model.

The reaction numbers correspond to those in Figure 1. The units of concentration in all the reactants are nanomolar [nM].

Reaction no.	Biochemical reactions
1	k_1 * [EGFR] * [EGF] - k_{-1} * [EGF/EGFR]
2	k_2 * [EGF/EGFR] * [EGF/EGFR] - k_{-2} * [E11]
3	k_3 * [E11] - k_{-3} * [E11P]
4	V_4 * [E11P] / (K_{m4} + [E11P])
5	k_5 * [E11P] * [Cbl] - k_{-5} * [E11P/Cbl]
6	k_6 * [E11P/Cbl]
7	k_7 * [E11P] * [GAP] - k_{-7} * [E11P/GAP]
8	k_8 * [E11P] * [Shc] - k_{-8} * [E11P/Shc]
9	k_9 * [E11P/Shc] - k_{-9} * [E11P/ShcP]
10	k_{10} * [E11P/ShcP] - k_{-10} * [E11P] * [ShcP]
11	V_{11} * [ShcP] / (K_{m11}+ [ShcP])
12	k_{12} * [E11P/ShcP] * [Grb2/SOS] - k_{-12} * [E11P/ShcP/Grb2/SOS]
13	k_{13} * [E11P/ShcP/Grb2/SOS] - k_{-13} * [E11P] * [ShcP/Grb2/SOS]
14	k_{14} * [ShcP/Grb2/SOS] - k_{-14} * [ShcP] * [Grb2/SOS]
15	V_{15} * [E11P/ShcP/Grb2/SOS] * [RasGDP] / (K_{m15} + [RasGDP])
16	V_{16} * [RasGTP] / (K_{m16} + [RasGTP])
17	V_{17} * [E11P/GAP] * [RasGTP] / (K_{m17} + [RasGTP])
18	V_{18} * [RasGTP] * [Raf1] / (K_{m18} + [Raf1])
19	V_{19} * [Raf1A] / (K_{m19} + [Raf1A])
20	V_{20} * [Raf1A] * [MEK] / (K_{m20} * (1.0 + [MEKP] / K_{m22}) + [MEK])
21	V_{21} * [PP2A] * [MEKP] / (K_{m21} * (1.0 + [MEKPP] / K_{m23}) + [MEKP])
22	V_{22} * [Raf1A] * [MEKP] / (K_{m22} * (1.0 + [MEK] / K_{m20}) + [MEKP])
23	V_{23} * [PP2A] * [MEKPP] / (K_{m23} * (1.0 + [MEKP] / K_{m21}) + [MEKPP])
24	V_{24} * [MEKPP] * [ERK] / (K_{m24} * (1.0 + [ERKP] / K_{m26}) + [ERK])
25	V_{25} * [MKP3] * [ERKP] / (K_{m25} * (1.0 + [ERKPP] / K_{m27}) + [ERKP])
26	V_{26} * [MEKPP] * [ERKP] / (K_{m26} * (1.0 + [ERK] / K_{m24}) + [ERKP])
27	V_{27} * [MKP3] * [ERKPP] / (K_{m27} * (1.0 + [ERKP] / K_{m25}) + [ERKPP])

Supplementary Table 2. Ordinary differential equations for the model.

Ordinary differential equations
$d[\mathrm{Cbl}] / dt = - v_5$
$d[\mathrm{Degradation}] / dt = + v_6$
$d[\mathrm{E11P/Cbl}] / dt = + v_5 - v_6$
$d[\mathrm{E11P/GAP}] / dt = + v_7$
$d[\mathrm{E11P/ShcP/Grb2/SOS}] / dt = + v_{12} - v_{13}$
$d[\mathrm{E11P/ShcP}] / dt = + v_9 - v_{10} - v_{12}$
$d[\mathrm{E11P/Shc}] / dt = + v_8 - v_9$
$d[\mathrm{E11P}] / dt = + v_3 - v_4 - v_5 - v_7 - v_8 + v_{10} + v_{13}$
$d[\mathrm{E11}] / dt = + v_2 - v_3 + v_4$
$d[\mathrm{EGFR}] / dt = - v_1$
$d[\mathrm{EGF/EGFR}] / dt = + v_1 - 2v_2$
$d[\mathrm{EGF}] / dt = 0$
$d[\mathrm{ERKPP}] / dt = + v_{26} - v_{27}$
$d[\mathrm{ERKP}] / dt = + v_{24} - v_{25} - v_{26} + v_{27}$
$d[\mathrm{ERK}] / dt = - v_{24} + v_{25}$
$d[\mathrm{GAP}] / dt = - v_7$
$d[\mathrm{Grb2/SOS}] / dt = - v_{12} + v_{14}$
$d[\mathrm{MEKPP}] / dt = + v_{22} - v_{23}$
$d[\mathrm{MEKP}] / dt = + v_{20} - v_{21} - v_{22} + v_{23}$
$d[\mathrm{MEK}] / dt = - v_{20} + v_{21}$
$d[\mathrm{MKP3}] / dt = 0$
$d[\mathrm{PP2A}] / dt = 0$
$d[\mathrm{Raf1A}] / dt = + v_{18} - v_{19}$
$d[\mathrm{Raf1}] / dt = - v_{18} + v_{19}$
$d[\mathrm{RasGDP}] / dt = - v_{15} + v_{16} + v_{17}$
$d[\mathrm{RasGTP}] / dt = + v_{15} - v_{16} - v_{17}$
$d[\mathrm{ShcP/Grb2/SOS}] / dt = + v_{13} - v_{14}$
$d[\mathrm{ShcP}] / dt = + v_{10} - v_{11} + v_{14}$
$d[\mathrm{Shc}] / dt = - v_8 + v_{11}$

Supplementary Table 3. Estimated kinetic constants for the H1299 models. Parameters which vary among the H1299 models are in blue.

	WT model<br>EGFR-WT model<br>L858R model B		L858R model A	
**Reaction no.**	***k, V***	***k_-, K_m***	***k, V***	***k_-, K_m***
1	1.04E-03	3.47E-01	1.04E-03	3.47E-01
2	2.41E-01	1.73E+00	2.41E-01	1.73E+00
3	1.24E+01	8.53E-04	1.85E+00	8.53E-04
4	8.21E+01	1.89E-02	8.21E+01	1.89E-02
5	4.08E-01	1.49E-01	1.63E-01	1.49E-01
6	2.90E-02	-----	2.90E-02	-----
7	6.75E-03	4.57E-05	2.70E-03	4.57E-05
8	1.35E+01	9.62E+00	5.39E+00	9.62E+00
9	1.07E+01	1.49E+01	1.07E+01	1.49E+01
10	5.48E+00	4.08E+01	5.48E+00	4.08E+01
11	3.48E+00	1.00E+01	3.48E+00	1.00E+01
12	1.79E+02	3.63E+01	1.79E+02	3.63E+01
13	4.38E+01	3.30E-01	4.38E+01	3.30E-01
14	1.70E-03	1.12E+01	1.70E-03	1.12E+01
15	6.72E-01	1.74E-02	6.72E-01	1.74E-02
16	4.11E-02	8.81E-01	4.11E-02	8.81E-01
17	5.67E-01	3.56E+01	5.67E-01	3.56E+01
18	1.92E+00	8.82E+01	1.92E+00	8.82E+01
19	4.38E-01	1.73E+01	4.38E-01	1.73E+01
20	2.30E-01	8.40E+01	2.30E-01	8.40E+01
21	4.47E-01	6.18E+00	4.47E-01	6.18E+00
22	5.32E-01	7.03E-02	5.32E-01	7.03E-02
23	5.06E-01	7.72E+00	5.06E-01	7.72E+00
24	6.31E+02	3.40E+03	6.31E+02	3.40E+03
25	5.06E-01	4.35E+02	5.06E-01	4.35E+02
26	5.22E-01	2.93E+01	5.22E-01	2.93E+01
27	5.98E-03	4.72E-02	5.98E-03	4.72E-02

Supplementary Table 4. Estimated initial concentrations of the cellular components. Parameters different among the H1299 models are in blue.

Reactants	WT model	EGFR-WT model<br>L858R model A	L858R model B
Cbl	2.56E+02	2.56E+02	5.12E+02
EGFR	2.36E+02	1.18E+03	1.18E+03
ERK	1.68E+02	1.68E+02	1.68E+02
GAP	1.66E+02	1.66E+02	1.66E+02
Grb2/SOS	1.90E+01	1.90E+01	1.90E+01
MEK	9.40E+02	9.40E+02	9.40E+02
MKP3	1.60E+01	1.60E+01	1.60E+01
PP2A	4.78E+01	4.78E+01	4.78E+01
Raf1	1.70E+01	1.70E+01	1.70E+01
RasGDP	4.02E+01	4.02E+01	4.02E+01
Shc	3.12E+02	3.12E+02	3.12E+02

[nM]

Supplementary Table 5. Search range for parameter estimation.

Lower and upper bounds were set to about one tenth of the minimum values and about ten times more than the maximum values of parameters used in the previous papers, respectively. Some estimated ranges were exceptionally set to be somewhat wide due to the lack of previous studies using the same equations as our model.

Species	Lower bound	Upper bound	References
Cbl	100	1000	Sasagawa *et al* (2005), Ung *et al* (2008)
EGFR	50	1000	Khodenko *et al* (1999), Wolf *et al* (2007)
ERK	100	1000	Hatakeyama *et al* (2003), Wolf *et al* (2007)
GAP	1	200	Wolf *et al* (2007)
Grb2/SOS	1	100	Hatakeyama *et al* (2003), Nakakuki *et al* (2008)
MEK	100	1000	Hatakeyama *et al* (2003), Birtwistle *et al* (2007)
MKP3	1	50	Hatakeyama *et al* (2003), Ung *et al* (2008)
PP2A	10	500	Hatakeyama *et al* (2003), Ung *et al* (2008)
Raf1	10	1000	Wolf *et al* (2007), Birtwistle *et al* (2007)
RasGDP	10	500	Nakakuki *et al* (2008), Ung *et al* (2008)
Shc	10	1000	Hatakeyama *et al* (2003), Birtwistle *et al* (2007)
k_1	exp(-10)	1000	Yamada *et al* (2004), Nakakuki *et al* (2008)
k_{-1}	exp(-10)	200	Hatakeyama *et al* (2003), Kiyatkin *et al* (2006)
k_2	0.0001	100	Schoeberl *et al* (2002), Yamada *et al* (2004)
k_{-2}	0.0001	500	Kiyatkin *et al* (2006), Nakakuki *et al* (2008)
k_3	0.005	100	Kiyatkin *et al* (2006), Nakakuki *et al* (2008)
k_{-3}	0.0001	5	Sasagawa *et al* (2005), Nakakuki *et al* (2008)
V_4	0.05	5000	Kholodenko *et al* (1999), Nakakuki *et al* (2008)
K_{m4}	exp(-10)	500	Kholodenko *et al* (1999), Nakakuki *et al* (2008)
k_5	0.01	5	Sasagawa *et al* (2005)
k_{-5}	0.01	5	Sasagawa *et al* (2005)
k_6	exp(-10)	0.5	Kiyatkin *et al* (2006), Birtwistle *et al* (2007)
k_7	exp(-10)	exp(10)	Schoeberl *et al* (2002), Ung *et al* (2008)
k_{-7}	exp(-10)	exp(10)	Schoeberl *et al* (2002), Ung *et al* (2008)
k_8	0.0001	500	Schoeberl *et al* (2002), Ung *et al* (2008)
k_{-8}	0.001	100	Ung *et al* (2008), Nakakuki *et al* (2008)
k_9	0.1	100	Hatakeyama *et al* (2003), Nakakuki *et al* (2008)
k_{-9}	0.001	50	Kholodenko *et al* (1999), Hatakeyama *et al* (2003)
k_{10}	0.0001	50	Yamada *et al* (2004), Kiyatkin *et al* (2006)
k_{-10}	exp(-10)	exp(10)	Khodenko *et al* (1999), Kiyatkin *et al* (2006)

V_{11}	0.001	20	Khodenko *et al* (1999), Hatakeyama *et al* (2003)
K_{m11}	10	3000	Khodenko *et al* (1999), Nakakuki *et al* (2008)
k_{12}	0.0005	500	Schoeberl *et al* (2002), Hatakeyama *et al* (2003)
k_{-12}	0.005	5000	Kholodenko *et al* (1999), Hatakeyama *et al* (2003)
k_{13}	exp(-10)	exp(10)	Hatakeyama *et al* (2003), Kiyatkin *et al* (2006)
k_{-13}	exp(-10)	exp(10)	Kholodenko *et al* (1999), Hatakeyama *et al* (2003)
V_{14}	exp(-10)	500	Hatakeyama *et al* (2003), Kiyatkin *et al* (2006)
K_{14}	exp(-10)	500	Schoeberl *et al* (2002), Kiyatkin *et al* (2006)
V_{15}	0.01	500	Hatakeyama *et al* (2003), Nakakuki *et al* (2008)
K_{m15}	0.01	2000	Hatakeyama *et al* (2003), Nakakuki *et al* (2008)
V_{16}	0.01	20	Hatakeyama *et al* (2003), Nakakuki *et al* (2008)
K_{m16}	0.005	200	Hatakeyama *et al* (2003), Nakakuki *et al* (2008)
V_{17}	0.01	20	Hatakeyama *et al* (2003), Nakakuki *et al* (2008)
K_{m17}	0.005	200	Hatakeyama *et al* (2003), Nakakuki *et al* (2008)
V_{18}	0.1	200	Hatakeyama *et al* (2003), Nakakuki *et al* (2008)
K_{m18}	exp(-10)	5000	Birtwistle *et al* (2007), Nakakuki *et al* (2008)
V_{19}	0.0001	200	Hatakeyama *et al* (2003), Birtwistle *et al* (2007)
K_{m19}	0.001	10000	Birtwistle *et al* (2007), Nakakuki *et al* (2008)
V_{20}	0.005	50	Hatakeyama *et al* (2003), Nakakuki *et al* (2008)
K_{m20}	0.01	5000	Birtwistle *et al* (2007), Nakakuki *et al* (2008)
V_{21}	0.005	30	Hatakeyama *et al* (2003), Ung *et al* (2008)
K_{m21}	1	exp(10)	Hatakeyama *et al* (2003), Ung *et al* (2008)
V_{22}	0.05	50	Hatakeyama *et al* (2003), Ung *et al* (2008)
K_{m22}	0.01	3000	Hatakeyama *et al* (2003), Ung *et al* (2008)
V_{23}	0.005	30	Hatakeyama *et al* (2003), Ung *et al* (2008)
K_{m23}	0.001	500	Hatakeyama *et al* (2003), Nakakuki *et al* (2008)
V_{24}	1	5000	Hatakeyama *et al* (2003), Nakakuki *et al* (2008)
K_{m24}	exp(-10)	exp(10)	Hatakeyama *et al* (2003), Nakakuki *et al* (2008)
V_{25}	0.005	50	Hatakeyama *et al* (2003), Ung *et al* (2008)
K_{m25}	0.001	2000	Hatakeyama *et al* (2003), Ung *et al* (2008)
V_{26}	exp(-10)	200	Hatakeyama *et al* (2003), Nakakuki *et al* (2008)
K_{m26}	0.005	exp(10)	Hatakeyama *et al* (2003), Nakakuki *et al* (2008)
V_{27}	0.005	5	Hatakeyama *et al* (2003), Ung *et al* (2008)
K_{m27}	0.001	500	Hatakeyama *et al* (2003), Ung *et al* (2008)

Supplementary Table 6. Dataset for parameter estimation.

		H1299WT			H1299EGFR-WT			H1299L858R		
		EGF 10nM	EGF 1nM	EGF 0.1nM	EGF 10nM	EGF 1nM	EGF 0.1nM	EGF 10nM	EGF 1nM	EGF 0.1nM
Phosphorylated EGFR	0 min	0.000	0.000	0.000	0.000	0.000	0.000	0.000	0.000	0.000
	1 min	0.051	0.034	0.004	0.667	0.126	0.000	0.254	0.063	0.004
	5 min	0.035	0.033	0.004	0.901	0.094	0.000	0.299	0.020	0.007
	10 min	0.037	0.026	0.003	1.000	0.081	0.000	0.316	0.067	0.005
	30 min	0.022	0.018	0.001	0.930	0.102	0.000	0.328	0.077	0.002
Phosphorylated Shc	0 min	0.000	0.000	0.000	0.000	0.000	0.000	0.000	0.000	0.000
	1 min	0.536	0.291	0.000	0.833	0.462	0.056	0.538	0.140	0.015
	5 min	0.430	0.314	0.013	0.962	0.410	0.015	0.607	0.084	0.011
	10 min	0.429	0.211	0.001	1.000	0.278	0.003	0.584	0.211	0.020
	30 min	0.137	0.115	0.009	0.711	0.213	0.000	0.437	0.210	0.005
Phosphorylated MEK	0 min	0.000	0.000	0.000	0.000	0.000	0.000	0.000	0.000	0.000
	1 min	0.238	0.025	0.000	0.448	0.353	0.093	0.316	0.054	0.000
	5 min	0.636	0.471	0.254	1.000	0.864	0.202	0.966	0.302	0.055
	10 min	0.201	0.286	0.006	0.836	0.387	0.025	0.681	0.313	0.012
	30 min	0.009	0.022	0.000	0.372	0.158	0.000	0.305	0.075	0.000
Phosphorylated ERK	0 min	0.000	0.000	0.000	0.000	0.000	0.000	0.000	0.000	0.000
	1 min	0.093	0.004	0.000	0.511	0.385	0.040	0.186	0.034	0.000
	5 min	0.755	0.644	0.359	0.891	1.014	0.409	0.656	0.460	0.067
	10 min	0.494	0.441	0.251	1.000	0.721	0.166	0.566	0.481	0.114
	30 min	0.264	0.170	0.000	0.900	0.440	0.000	0.465	0.211	0.000
